# Viability Is Associated with Melanin-Based Coloration in the Barn Swallow (*Hirundo rustica*)

**DOI:** 10.1371/journal.pone.0060426

**Published:** 2013-04-03

**Authors:** Nicola Saino, Maria Romano, Diego Rubolini, Roberto Ambrosini, Manuela Caprioli, Aldo Milzani, Alessandra Costanzo, Graziano Colombo, Luca Canova, Kazumasa Wakamatsu

**Affiliations:** 1 Department of Biosciences, University of Milan, Milan, Italy; 2 Department of Biotechnology and Biosciences, University of Milano-Bicocca, Milan, Italy; 3 Dipartimento di Biologia e Biotecnologie, Università degli Studi di Pavia, Pavia, Italy; 4 Department of Chemistry, Fujita Health University School of Health Sciences, Toyoake, Aichi, Japan; University of Lausanne, Switzerland

## Abstract

Pigmentation of body surface in animals can have multiple determinants and accomplish diverse functions. Eumelanin and pheomelanin are the main animal pigments, being responsible of yellow, brownish-red and black hues, and have partly common biosynthetic pathways. Many populations of vertebrates show individual variation in melanism, putatively with large heritable component. Genes responsible for eu- or pheomelanogenesis have pleiotropic but contrasting effects on life-history traits, explaining the patterns of covariation observed between melanization and physiology (e.g. immunity and stress response), sexual behavior and other characters in diverse taxa. Yet, very few studies in the wild have investigated if eu- and pheomelanization predict major fitness traits like viability or fecundity. In this correlative study, by contrasting adult barn swallows (*Hirundo rustica*) matched for age, sex, breeding site, and year and date of sampling, we show that males but not females that survived until the next year had paler, relatively more eu- than pheomelanic pigmentation of ventral body feathers. Better performance of individuals that allocate relatively more to eumelanogenesis was expected based on previous evidence on covariation between eumelanic pigmentation and specific traits related to immunity and susceptibility to stress. However, together with the evidence of no covariation between viability and melanization among females, this finding raises the question of the mechanisms that maintain variation in genes for melanogenesis. We discuss the possibility that eu- and pheomelanization are under contrasting viability and sexual selection, as suggested by larger breeding and sperm competition success of darker males from other barn swallow subspecies.

## Introduction

Coloration is a major target of natural selection as it can influence thermoregulation, and camouflage of prey and their predators, and mediates social and sexual communication. Melanins are the most common pigmentary determinants of animal coloration [1], [2] and are synthesized starting from their common precursor, L-tyrosine, through oxidation and decarboxylation reactions that result in either of two biosynthetic endpoints: pheomelanin, which is responsible for yellow to reddish-brown hues, and eumelanin, which is responsible for grey to black hues [3]–[6]. However, in most cases both melanin forms are produced concomitantly, resulting in ‘mixed melanin’ pigmentation whereby perceived color depends on both absolute and relative concentrations of either melanin form [7], [8].

Several observational and experimental studies on model species or genetically engineered strains under artificial conditions have shown that melanogenesis and thus melanin-based pigmentation covary with a number of traits ranging from regulation of metabolism and energy homeostasis, to immunity, response to stressors mediated by the hypothalamic-pituitary-adrenal axis, social aggressiveness and sexual behavior [9]. Eumelanic coloration, in particular, has been shown to positively covary with traits related to social and sexual performance, resistance to stress, immunity and metabolism in wild vertebrates [9]. Detailed studies of the very few wild bird populations that have been investigated so far have provided consistent evidence for an association between physiological and behavioral traits and feather melanization [10]–[15], while information from other free-ranging vertebrates is sparse.

At the mechanistic level, covariation between eu- or pheomelanogenesis and fitness traits has been hypothesized to be enforced by the pleiotropic effects of genes that code for melanocortins and their inverse agonist and antagonist, the agouti-signalling protein (ASIP). Binding of melanocortins to the melanocortin-1 receptor (MC1R) at e.g. hair and feather follicles triggers eumelanogenesis, whereas binding of ASIP blocks eumelanogenesis and triggers pheomelanogenesis [6], [9]. However, melanocortins can also bind to other (MC2-5) receptors which are expressed in other target cells, thus controlling a broadly diverse set of physiological and behavioral traits and causing such traits to covary with melanization. Because of pleiotropic, antagonistic effects of genes for eu- or pheomelanogenesis, opposite associations between eu- vs. pheomelanization and other target traits may be expected [16].

Such multi-faceted associations with fitness traits suggest that melanin-based colorful traits, and feather colors of many bird species in particular, may have evolved under the influence of inter- as well as intra-sexual selection for signals that reflect variation in inherent quality among individuals [16]–[19]. Variation in melanin-based colorations may advertise the genetic/phenotypic quality to same-sex competitors for mating opportunities or to choosy opposite-sex mates. Alternatively, melanin-based coloration may reflect individual differences in life-history strategies that are associated with minor or no difference in fitness. This could be the case because the genes that control eu- and pheomelanogenesis target partly different traits. In addition,they may cause physiological tradeoffs among the traits that they pleiotropically affect.

Independently of any actual signaling role, however, identifying any association between melanization and fitness traits in populations under a natural sexual and natural selection regime is pivotal to any study of the evolution of melanism. Yet, while several studies on a very limited set of bird species have investigated the association between feather melanization and physiological performance at specific traits in adults or offspring before maturity [10], [15], [20]–[23], analyses of major fitness components such as fecundity or viability are almost lacking [24], [25].

In this correlational study we tested whether viability is associated with melanin-based coloration in a long-distance migratory, colonial, socially monogamous passerine bird, the barn swallow (*Hirundo r. rustica*). In our study population, melanin-based color of belly feathers ranges from white to brownish [26]. Increasing concentrations of both eu- and pheomelanin in belly feathers of males and females produce darker brownish, more saturated color in our study population and also in a North American population of the subspecies *H. r. erythrogaster* [27]. The relative concentrations of pheo- and eumelanin (pheo:eu ratio) is significantly larger in darker belly feathers of males [26], [27]. In females, the relationships between pheo:eu ratio and color have the same sign as in males but are statistically non-significant [26]. Hence, males with paler, whitish belly feathers have significantly more saturated and *relatively* more eu- than pheomelanic pigmentation, and a consistent, though weaker pattern of association between *relative* concentration of melanin forms and color is observed for females.

Color of belly feathers was measured by spectrometry while taking cone sensitivity of passerines into account [28]. We compared color of individuals that did not survive until the following breeding season with color of individuals that survived for one year, matched for year, sex, age, as well as breeding colony and capture date. This paired sampling design allowed us to control for potentially confounding genetic (sex), ontogenetic (age), and environmental (year, breeding colony) effects, as well as for any variation in condition as reflected in arrival and thus capture date.

Larger allocation to eu- than pheomelanogenesis is associated with better performance at a number of life-history traits (see above). We therefore expected paler, relatively more eumelanic individuals to be more viable than darker, relatively more pheomelanic ones. Because the finding of an association between color and relative pheo- and eumelanization was less stringent for females than males, we expected the relationship between color and viability to be weaker in females. Because little is known on age-related variation of belly feathers color in barn swallows, we had no predictions on age-related variation in the association between color and survival.

## Methods

### Field Procedures

We studied adult barn swallows breeding in 10 colonies ( = farms) located near Milano (N Italy) in 11 years over the period 1997–2010. In every year in this period we did repeated capture session throughout the breeding seasons (March-August) and individually marked with numbered metal rings the adults at the rural building where they breed. Adult barn swallows spend the night inside the rooms (mainly cowsheds) where they nest. We could therefore effectively capture the vast majority of the breeding individuals by placing mist-nest at all the exits before dawn. Because every year we marked the vast majority of the breeding adults, and barn swallows in our study area show extremely high breeding philopatry (i.e. adults do not move to a different colony to breed in consecutive years) and very low natal philopatry, the unmarked swallows that were captured in any particular year in a farm where we had captured the adults in the previous year could be confidently assumed to be 1-year old individuals, and age could therefore be assign at recaptures at the same colony in the following years (see [29]–[31] and references therein). Because of extremely high breeding philopatry, adults that did not return to their breeding colony in a given year could be assumed to have died [30], [31]. At the first capture in any particular year we plucked 2–3 feathers from the left, paramedial belly plumage region. The feathers we collected are produced at the time of the winter molt in sub-Saharan Africa [32]. The collected feathers were stored in small plastic bags and kept at room temperature in a dark box until color analysis.

Survival could be affected by several extrinsic factors: depending on ecological conditions during migration and wintering it could vary among years; it could change due to senescence or in a sex-dependent way; moreover, it could vary among colonies if ecological conditions vary among breeding sites. In addition, although the sources of variation that specifically operate in barn swallows are unknown, it can be expected that color may vary according to genetic (sex) [1], ontogenetic (age) [33], [34] and environmental (colony and year) [35] factors. Because our aim was to test for a covariation between survival until the next breeding season and color, we planned our analyses in order to optimally control for these potentially confounding sources of variation in both mortality and color by comparing individuals that survived until the following year (survivors) and individuals that did not survive (non-survivors) while controlling for variation in traits that may differentially affect survival, and that differed only in survivorship until the following year. In particular, for each individual included in the present study that survived from year *i* to year *i*+1, but not until year *i+*2 (i.e. survivors were included only when in their penultimate year of life, see below) we identified an individual of the same year, age, sex, and, whenever possible, colony and first capture date (see below), that did not survive until year *i*+1. By including survivors only in their penultimate year of life we avoided matching long-lived survivors in their first year(s) of life with short-lived non-survivors in their last year of life. Matching of individuals with the same (or similar) first capture date allowed us to compare individuals sharing similar general conditions and that bred under similar ecological conditions. When two or more individuals fulfilled these criteria, one of them was randomly chosen. While the year, age, sex and survivorship criteria for matching were always fulfilled, matching by farm/first capture date was not feasible for just 9% of the non-survivors. In these cases, we choose an individual from another farm with the most close first capture date. No significant among-farms difference in color existed in the present sample.

When more than one individual matched the above criteria, we choose the individual with the sequential identity number (that we gave to all individuals according to their order of capture), closer to that of the focal surviving individual and, when needed, we alternated between the individual with the closer higher and lower identity number. We emphasize, however, that because pairing of individuals was done blind of the color of the feathers, collection of the feathers occurred years before color measurement and different persons did the pairing or, respectively, collected the feathers, we are fully confident that the paring procedure did not introduce any bias.

### Color Measurement

Color of belly feathers considerably varies among individuals from white to brownish [26]. Color was quantified by spectrophotometry of one, randomly chosen belly feather using an Avantes DH-2000 spectrometer in a dark chamber. Illumination was provided by a combined deuterium-tungsten halogen source. Reflectance of the samples was always referred to white and black standards. Two reflectance spectra were obtained from each feather by illuminating a 2.5 mm^2^ field, centered approximately 2.5 mm from the distal end of the feather. Reflectance spectra were processed using the tetrachromatic color space model [36] by means of the TetraColorSpace program (Version 1a; [28]) run in MATLAB 7 (MathWorks, Natick, MA). This procedure has the advantage of incorporating information on both plumage reflectance spectra and bird cone sensitivity functions, thus allowing us to realistically describe color as perceived by birds. We assumed UVS cone type-retina and used spectral sensitivity of the blue tit (*Cyanistes caeruleus*) because this is the species more phylogenetically close to the barn swallow for which spectral sensitivity information is implemented in TetraColorSpace program. In the tetrahedral color space model, color is described by the θ and φ components, which represent hue in the red-green-blue (θ) or in the ultraviolet (φ) spectra, and by ‘achieved chroma’ (rA), which is a measure of color saturation. In the range of variation of barn swallow belly feathers color, increasing values of θ indicate paler, less brownish coloration. Increasing value of rA indicate increasing color saturation. No verbal description of variation in φ can be provided because φ mainly represent color in the UV band.

Repeatability of the color variables, as estimated by measuring twice the same feather, is known to be large (>0.73; n = 45 individuals). Among-feathers repeatability estimated by measuring two different feathers from the same region was also larger than 0.74 for all variables (n = 10 individuals for both plumage regions). In the analyses, we thus used the color variables values averaged between the two reflectance measurements obtained from the same individual.

### Statistical Analyses

Because of the paired sampling protocol (see above), the association between survival and color variables was investigated by one-sample t-tests where we tested if the difference in color variables values between matched individuals (survivor minus non-survivor) differed from 0. To test whether the difference in color between survivors and non-survivors varied according to age, sex or their combined effects, we computed the differences in color variables and analyzed them in linear mixed models where year was always included as a random factor. Colony (either of the survivor or non-survivor in the 9% of the cases where observations could not be matched by site) was also initially included as a random factor in the models, but it did not significantly contribute to the fitting of the mixed models (details not shown) and was therefore removed. The effect of the random factors was tested by Wald Z tests. Age at color measurement ranged between 1 and 6 years. Because the frequency of 5- and 6-years old individuals was small, the 4–6 years old individuals were lumped in the same class and age was considered as a four levels factor (age: 1, 2, 3, ≥4 years). Standardized selection differentials for color variables were estimated as (µ_a_ – µ_b_)/σ(b), where x_a_ is the mean phenotypic value of the color variable of the individuals that survived, σ _b_ is the mean phenotypic value before selection (i.e. among survivors and non-survivors pooled), and SD(b) is the standard deviation of the color variable before selection.

### Ethics Statement

Upon capture, barn swallows were kept in cloth bags in a safe position not accessible to predators, as is standard practice in bird ringing studies. All individuals were sexed and released as soon as possible, usually within 1 hour of capture. After being released, swallows behaved normally and observations at the nest on hundreds of individuals confirmed that they resumed their normal breeding activities. The study was carried out under ringing permit 0665 released by the Istituto Nazionale per la Fauna Selvatica, which issues all the relevant permissions required. No approval from an ethics committee is currently required for this kind of study according to the existing legislation.

## Results

**Figure 1:**
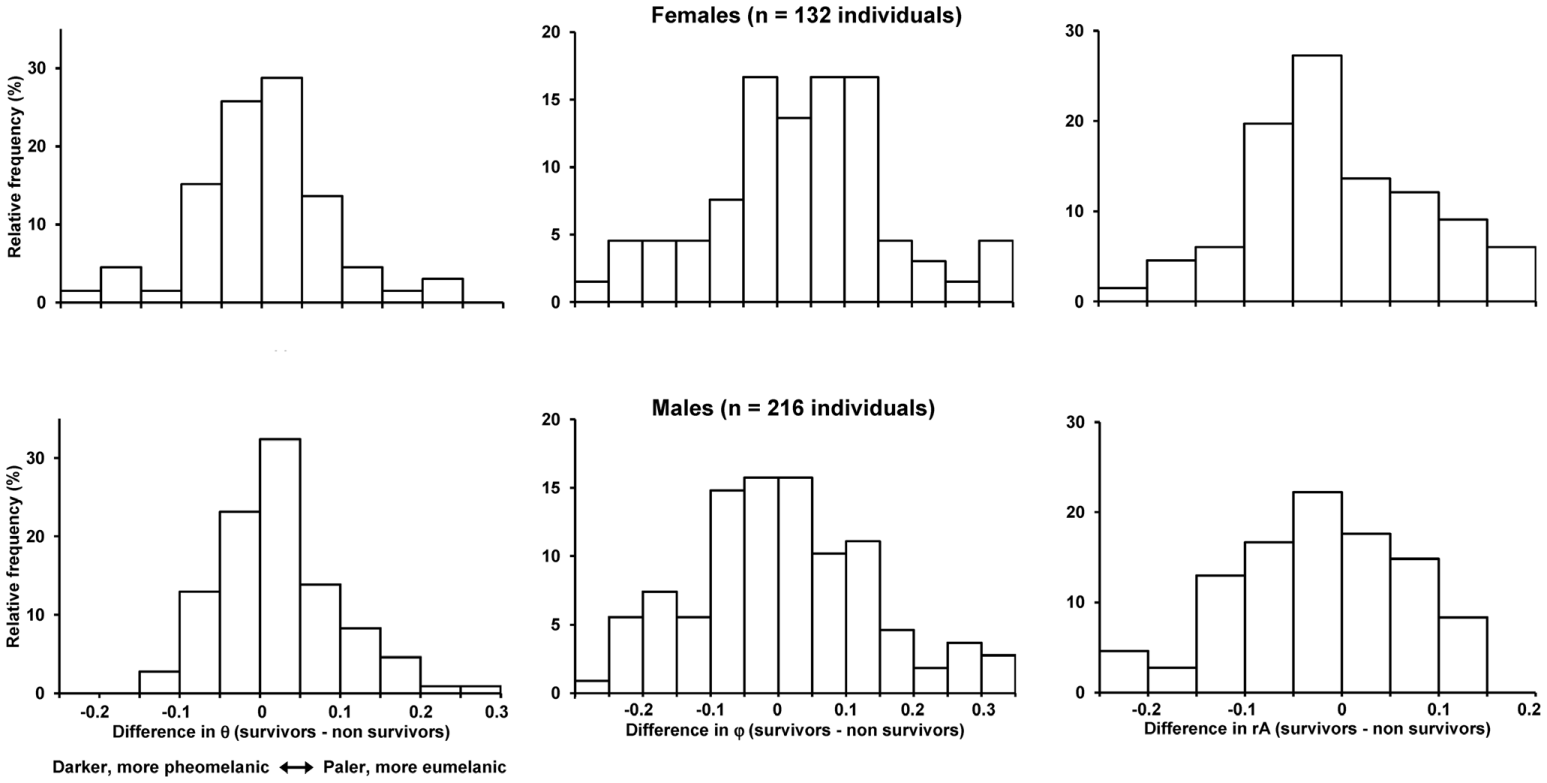
Frequency distribution of the difference in tetrahedral color components between female (upper panels) or male (lower panels) barn swallows that survived or did not survive from one breeding season to the next. Each survivor was matched to a non-surviving individual of the same sex and age, sampled in the same year and, whenever possible (91% of the cases), breeding colony and capture date (see Methods).

We measured tetrahedral color components of belly feathers from 216 males and 132 females organized in pairs of individuals that did or did not survive until the following breeding season. The difference (survivor minus non-survivor) of color variables within pairs of males significantly deviated from 0 for θ and rA, but not for φ (Table 1; Fig. 1). In particular, θ was significantly higher among survivors, indicating that survivors had paler, relatively more eumelanic coloration than non-survivors. In addition, rA, indicating color saturation, was significantly smaller among survivors than non-survivors (Fig. 1). Conversely, none of the differences in coloration components significantly deviated from 0 within pairs of females, although mean φ of survivors was marginally non-significantly larger than mean φ of the non-survivors (Table 1; Fig. 1).

**Table 1:** Mean (SE) tetrahedral color component values of males or females that did or did not survive.

	Males				Females			
	Mean (SE)		t_107_	P	Mean (SE)		t_65_	P
Color component	Surviving	Non-surviving			Surviving	Non-surviving		
θ	0.163 (0.007)	0.141 (0.006)	3.08	0.003	0.172 (0.009)	0.169 (0.010)	0.32	0.749
φ	−0.959 (0.014)	−0.971	0.80	0.425	−1.003 (0.016)	−1.036 (0.011)	1.99	0.051
rA	0.223 (0.007)	0.246 (0.006)	−2.70	0.008	0.214 (0.008 =	0.221 (0.008)	−0.65	0.520

Surviving and non-surviving individuals were matched for year and age and, whenever possible (91% of the cases), for breeding site and capture date. Increasing values of θ indicate paler, relatively more eumelanic plumage. Significance of the deviation of mean differences between surviving and non-surviving individuals from zero was assessed by *t*-tests. Sample sizes are 108 pairs of males and 66 pairs of females.

The relative frequency (%) of males (and thus of pairs of males) in the four age classes was: age 1 yr: 33.3; age 2∶32.1; age 3∶16.5; age ≥4∶18.3. The corresponding age-specific frequencies for females were: 31.8, 25.8, 25.8, 16.7. Mixed models with year as a random factor disclosed no significant age by sex effects on the within-pairs differences in color (Table 2). In addition, no main effects of age emerged by excluding the interaction term, indicating that the difference between survivors and non-survivors in feather color did not differ among short- or long-lived birds. The difference in θ between survivors and non-survivors was larger among males than females, while the differences in φ or rA did not differ between sexes (Table 2; Fig. 1). ). No significant random effect of year emerged (P>0.216 in all models).

**Table 2:** Linear mixed models of the difference in tetrahedral color components between survivors and non-survivors in relation to sex and age, with year as a random factor.

	θ			φ			rA		
	F	df	P	F	df	P	F	df	P
Sex	5.34	1,149	0.020	0.37	1,147	0.543	1.53	1,169	0.218
Age	1.74	3,164	0.160	0.46	3,163	0.712	0.68	1,169	0.568
Sex×Age	0.74	3,161	0.529	1.31	3,161	0.274	1.03	3,166	0.381

F-statistics for the main effects of sex and age are obtained from a mixed model excluding the interaction term (see also Results).

Estimated selection differentials on θ and rA of males were 0.166 and 0.162, respectively.

## Discussion

Variation in coloration among conspecific individuals is commonplace in diverse vertebrate taxa [1]. In birds, large variation is known to occur in melanin-based coloration even among same-sex individuals from the same population. Yet, the genetic, ontogenetic and environmental sources of variation in melanin-based coloration, the mechanisms that maintain color polymorphism, and the fitness correlates of such variation are poorly known.

Melanin-based color of belly feathers of barn swallows widely varies, from white to brownish, among individuals from the same geographical population and also, at a large spatial scale, among geographical populations from distant regions or different continents [37]–[39]. In this study we found that individual males that survived from one breeding season to the next had less brownish (higher θ) and less saturated (lower rA) color than males that did not survive. Our sample included a large number of pairs of surviving and non-surviving individuals that were matched for sex, age, year of sampling and, in the vast majority of the cases, also for breeding colony and date of capture. Hence, our result was unaffected by sex and ontogenetic or environmental confounding effects. However, the same rigorous sampling design did not disclose any significant difference in color components among females. Indeed, while the size of the sample was considerably smaller for females than males, the tests on θ and rA were far from statistical significance. In addition, the difference in color between surviving and non-surviving individuals did not depend on age.

Color of belly feathers in the same barn swallow population depends on the concentration of both eu- and pheomelanin: larger concentrations of both melanin forms are associated with more saturated, darker brownish hues, and with larger values of the φ tetrahedral color component, mainly accounting for UV reflectance [26]. Since a larger pheo- to eumelanin ratio is associated with darker brownish, more saturated color, the present results show that males that did not survive till the next breeding season had allocated relatively more to pheo- compared to eu-melanization. This result is consistent with the expectations because the *relative* levels of eu- or pheomelanization may depend on the balance between the effects of antagonistic genes that trigger synthesis of either melanin form, and individuals that allocate more to eu- than pheomelanogenesis have been shown to have better performance at several functions (e.g. immunity, stress resistance, see [9]. Differential survival according to color may result from predation effects. In the present case, however, given the behavior of the most common predators of barn swallows [38], it is difficult to imagine that such color-based prey selectivity occurs and that color-related predation risk occurs among males but not females. An alternative, though as yet untested interpretation is that resistance to food deprivation varies with coloration [40], [41].

Viability selection differentials for the θ and rA color components were quite large (ca. 0.165). Plumage color in the barn swallow as well as in other bird species has been found to have large heritability (e.g. [10], [26]), though with some important exceptions [35], [42], implying the existence of large additive genetic variance and/or small environmental variation. A question therefore arises as to which mechanisms maintain heritable color variation in the presence of viability selection, as selection on color components seemed not to vary among years.

The barn swallow is a short-lived bird with a life-expectancy at sexual maturation of 1–2 years [31], [37], [38]. This implies that a large fraction of the adults have just one breeding season, normally with 1–2 broods [37] (our unpublished data). Fledging success is high and variance in brood size is relatively small [37], [39]. Hence, survival is a major determinant of lifetime fitness both via an effect on the lifetime number of breeding episodes and via the positive effect that relatively early breeding by two or more years old individuals has on offspring longevity [31]. However, in our study population sperm competition is intense, and 30–40% of the offspring are sired by a male different from their social father [43], [44]. Thus, variation in breeding success realized by males in terms of offspring in their own broods and offspring resulting from extra-pair fertilizations may partly or wholly compensate for reduced longevity. No information is yet available from our study population on the relationship between color and breeding success or sperm competition. In other barn swallow populations (North America, East Mediterranean region) [45], [46] it has been shown that color predicts breeding or sperm competition success, with darker males having larger paternity (proportion of biological offspring) in their broods or larger breeding output. Taken together, these pieces of evidence may suggest that paler, more eumelanic individuals are more viable but realize smaller reproductive success per breeding season because, for example, of the larger levels of cuckoldry that they experience. In this scenario, genetically based color variation may be maintained by contrasting sexual and viability selection. However, it must be emphasized that geographical barn swallow populations have been shown to markedly differ in several aspects of morphology, coloration and behavior, including sexual selection [37], [47], [48], and more information on the fitness correlates of darkness in belly coloration from our study population is required before any conclusion can be drawn. It should also be noted that selection in relation to color may also occur during the first year of life [25], although this hypothesis will be difficult to test owing to practical difficulties set by natal dispersal.

Contrary to males, we observed no significant association between feather color and annual survival among females. In fact, in females there was only a marginally non-significant hint to a difference in UV hue (φ) between survivors and non-survivors, while the difference in visible color (θ) and saturation was far from significant. In our study population, females have smaller concentrations of both pheo- and eumelanin compared to males in belly feathers [26]. Although, as observed in males, pheo- and eumelanin concentrations predict color, the ratio between pheo- and eumelanin concentration is weakly correlated with tetrahedral color variables. Hence, females may adopt different coloration strategies compared to males and weak covariation between relative allocation to pheo- or eumelanogenesis may result in poor association between color and viability.

Color of throat feathers of barn swallows has been shown to change with age [34]. However, in this study we observed no variation in the difference between color of surviving and non-surviving individuals according to age, implying that viability selection on color does not vary with age.

In conclusion, we showed that color variation in males but not females of the migratory barn swallow is associated with viability. Individuals with paler, relatively more eu- than pheomelanic color had larger viability, as predicted based on the general observation from diverse taxa of better performance at several life-history traits by eumelanic individuals. Viability selection might be compensated by contrasting sexual selection pressures if advantages of darker individuals in e.g. sperm competition observed in other populations operate also in our study population, thus promoting maintenance of heritable variation in color.
